# Decision-making and attention deficit hyperactivity disorder: neuroeconomic perspective

**DOI:** 10.3389/fnins.2024.1339825

**Published:** 2024-10-23

**Authors:** Aisha Sanober Chachar, Mahnoor Yousif Shaikh

**Affiliations:** ^1^Synapse, Pakistan Neuroscience Institute, Karachi, Pakistan; ^2^Dow Medical College, Dow University of Health Sciences, Karachi, Pakistan

**Keywords:** neuroscience and decision, ADHD, decision-making, neuroeconomics, delay discounting in ADHD

## Abstract

The decision-making process involves various cognitive procedures influenced by the interplay between cognition, motivation, and attention, forming a complex neural framework. Attention is a fundamental cognitive element within decision-making mechanisms, and one of the conditions affecting the attentional system is attention deficit hyperactivity disorder (ADHD). Decision-making impairments in ADHD have significant economic consequences, necessitating effective policies and interventions to address this critical issue. Research from computational models and neuroscience suggests how cognitive functions’ workings and problems affect decision-making and provide insights into the neural implications of decision-making. This article explores the intersection of decision-making, ADHD, and neuroeconomics, highlighting research gaps, potential contributions, and implications for future policies.

## 1 Introduction

### 1.1 ADHD

Attention Deficit Hyperactivity Disorder (ADHD) is a lifelong neurodevelopmental disorder with a childhood-onset where up to 85% of individuals continue to experience the symptoms throughout their lives (Willcutt et al., 2012). The three clinical presentations of ADHD are inattentive, hyperactive-impulsive, or combined, based on the cluster of symptoms. These subsets of the disorders have their unique neurobiological basis that leads to specific symptoms and impact on one’s life. Such alterations within the orbitofrontal cortex (OFC) lead to problems with impulsivity and/or hyperactivity. Inadequate tuning of the dorsal anterior cingulate cortex (DACC) and dorsolateral prefrontal cortex (DLPFC) can lead to sustained or selective attentive symptoms, respectively. Research also suggests that abnormalities in the orbitofrontal-limbic circuits are affected among children with conduct disorder (Gao et al., 2020).

In contrast, irregularities in the dorsolateral prefrontal cortex are seen in children with sustained attention problems, which is associated with academic impairment, low self-esteem, adverse occupational outcomes, and lower adaptive functioning. Hyperactive-impulsive symptoms are associated with peer rejection, aggression, risky driving behaviors, and accidental injuries. The manifestation of ADHD can differ among age groups, with symptoms persisting into adolescence and adulthood in approximately 60% of children with ADHD (American Psychiatric Association, 2000; Table 1).

**TABLE 1:** ADHD differences according to age.

Stage	Presentation
Pre-school	Play < 3 min, not listening to “whirlwind,” no sense of danger
Primary school	ACTIVITIES < 10 min, forgetful, distracted, restless, intrusive, disruptive
Adolescence	Less persistent than others > 30 min, lack of focus/planning, fidgety, reckless
Adult	Details need to be completed, feels restless, forgotten appointments, impatience, accidents.

ADHD is a condition that can hinder cognitive functions, emotional regulation, and interpersonal relationships. Individuals diagnosed with ADHD experience symptoms of inattention, hyperactivity, and impulsivity, which can prove challenging. The existing literature indicates that ab dopamine pathways play a crucial role in the manifestation of ADHD symptoms. Specifically, the alterations affect reward anticipation, time perception, and emotional regulation. Additionally, ADHD exhibits a genetic predisposition, and individuals with a family history of the disorder are at a higher risk of developing it. Other risk factors include prenatal tobacco and alcohol exposure, prematurity, low birth weight, maternal emotional status during pregnancy, and low-level lead exposure. Several factors, such as family history, comorbidity, and adversity, can predict the long-term prognosis of ADHD. Individuals with ADHD often have other health conditions or disorders; for instance, adults with ADHD are more likely to receive a diagnosis of antisocial personality traits and substance abuse disorder. Children with ADHD have a learning disability about half the time and a conduct disorder about one in four times. Other disorders like anxiety, mood disorders, and learning disabilities are also frequently seen with ADHD.

ADHD is a significant public health issue that sets a considerable economic burden globally (Doshi et al., 2012). These societal costs associated with ADHD are linked to one’s daily life in the form of academic underachievement, substance abuse, antisocial behavior, and higher rates of accidents and hospitalizations. Children with ADHD are more likely to experience learning difficulties, school absences, problematic relationships with peers, and a higher rate of ambulatory medical visits. As adults, people with ADHD face lower earnings and more reliance on social help. This leads to a 33% drop in earnings and a 15% rise in social assistance use (Fletcher, 2014). Children with ADHD are at risk of dropping out of school, becoming pregnant as teenagers, and committing criminal behavior (Ek et al., 2011; Harpin et al., 2016; Meinzer et al., 2020). Untreated ADHD increases the risk for future complications such as poor academic performance and learning delay, low self-esteem, poor social skills, and increased susceptibility to physical injury in childhood.

Assessing the economic burden of ADHD is a complex task. The manifestation of impairments varies between the different types of ADHD, but the severity of symptoms depends on the individual’s context (Tamm, 2009) making the economic burden of ADHD multifactorial. Medical and psychiatric comorbidities in adult patients with ADHD are primary drivers of the direct healthcare cost. It includes direct costs such as medication and therapy, indirect costs such as caregiver strain and missed work, and intangible costs such as marital stress, special education needs, tutoring costs, and in some cases, property damage, and for some parents paying and then showing up to extracurricular activities. Younger populations with ADHD may face increased accident rates, leading to vehicle damage costs and legal fees, and some families may face financial burdens resulting from legal issues related to delinquency.

Despite the variability among children diagnosed with the disorder and the challenges involved in diagnosis, ADHD has good clinical validity, meaning that impaired children share similarities, exhibit symptoms, respond to treatment, and are recognized with general consistency across clinicians (Nigg, 2006). It is noteworthy that the accuracy of diagnosing mental illnesses with coexisting conditions (comorbidities) can be insufficient, leading to severe consequences for patients. For instance, healthcare providers may treat patients with significant behavioral comorbidities for antisocial behavior instead of ADHD, thus overlooking ADHD. Additionally, there is a group of children with preliminary ADHD symptoms that do not meet the full criteria, leading to inaccurate diagnosis and treatment. While there is no evidence for preventing ADHD or conduct disorder, it is crucial to develop prevention techniques. Treating juvenile violence can impact related conditions like substance misuse. Combining medication with therapy can treat many comorbid illnesses, considering the child’s specific symptoms. The enormous economic burden of ADHD on both individuals and society, coupled with its negative impact on cognitive and socio-emotional development, warrants further research to understand its etiology, improve its identification, and develop more effective treatments (Hodgkins et al., 2011).

## 2 Current research gap

ADHD, in most cases, impacts decision-making significantly, affecting academic, occupational, and social functioning. Although existing research indicates various brain regions and neurotransmitter systems, a deeper understanding of the specific neural circuits and interactions underlying these connections is necessary. Longitudinal studies tracking the developmental trajectories of decision-making abilities in individuals with ADHD across the lifespan can help us identify critical periods of vulnerability and potential windows for intervention. Further research into how ADHD affects risk assessment and reward processing during decision-making tasks is needed, along with developing and testing interventions that enhance neuroplasticity and target specific neural circuits linked with decision-making impairments. Research should also account for individual differences, comorbid conditions, and variations in neural processes involved in ADHD, such as executive dysfunction and emotional dysregulation, and study their real-world implications, such as academic achievement, occupational success, and social functioning (Camerer, 2003; Derosiere and Duque, 2020; Glimcher and Fehr, 2013; Pastor et al., 2008; Smith and Price, 1982; Tecilla et al., 2022). Addressing these research gaps will advance our current knowledge of the neurobiological basis of decision-making impairments in ADHD and guide us to develop innovative and effective interventions that improve outcomes for individuals with ADHD.

## 3 Contribution

Neuroeconomic insights offer a novel perspective on human behavior, particularly regarding risk and reward dynamics. This understanding can enrich behavioral interventions, such as Cognitive Behavioral Therapy (CBT), by integrating neuroeconomic principles. By aiding patients in recognizing and modifying maladaptive decision-making patterns, clinicians can provide more effective support in navigating life’s challenges. Additionally, utilizing a translational approach to neuroeconomics can contribute to the nosological classification of ADHD. Understanding the neural substrates underlying decision-making processes within the framework of neuroeconomics can inform the diagnostic criteria and treatment strategies for ADHD. By bridging insights from neuroscience, psychology, and economics, the translational approach enhances our comprehension of ADHD and informs more targeted interventions.

## 4 Decision-making

The decision-making process involves several crucial steps that comprise problem recognition, data collection, option analysis, weighing pros and cons, selection of the best course of action, execution, and result evaluation. Making effective decisions involves analyzing different factors using either rational or intuitive approaches, depending on the complexity of the situation and one’s cognitive processes. Rational decision-making entails analyzing all available information, while intuitive decision-making relies on gut feelings and past experiences. Intuition is influenced by emotions and pattern recognition, which can lead to biases and judgment errors. Therefore, striking a balance between intuitive decisions and rational thinking is crucial for making well-informed choices in various situations.

Decision-making abilities can improve over time due to neuroplasticity, a process through which experiences refine neural pathways. The neural processes of the Prefrontal Cortex (PFC) and hippocampus are responsible for processing sensory input, retrieving complementary information, and making final decisions (Camerer et al., 2005). Similarly, external factors such as social context and cultural norms significantly influence decision-making processes. It is also to be kept into consideration that disorders such as addiction, ADHD, OCD, and certain types of dementia can disrupt decision-making processes by affecting specific neural pathways or causing conflicts among valuation systems (Rogers, 2011).

## 5 Decision-making in ADHD and developmental perspective

Understanding and assessing decision-making from a developmental standpoint is crucial. This is especially true for understanding children’s cognitive abilities at different ages. Children’s risk preferences may vary depending on social and economic factors, with education level a predictor of cognitive and academic abilities. Research on children’s decision-making in risky situations involves selecting options with varying potential outcomes and probabilities. Distinguishing between random and non-random outcomes is essential for accurate risk estimation, a skill even children as young as four can show. However, adolescents with ADHD may be particularly vulnerable in decision-making, necessitating vigilance against common pitfalls such as hasty judgments and disregarding conflicting evidence. Behavioral economic approaches consider individuals to be active decision-makers who choose based on the expected value of the alternatives. In the normative expected utility framework, individuals determine the expected value of a risky alternative by weighting its subjective potential payoff by probability. Rational decision-makers choose the option with the highest expected value. Their risk attitude influences their choice. This ranges from risk aversion to risk-seeking. Research shows that people with ADHD may not seek risks. They see the outcomes of risky behaviors as especially appealing or less dangerous. Besides, children with ADHD often underestimate the consequences of risky activities and overestimate their physical abilities while neglecting negative consequences. Adolescents with ADHD may have lesser negative expectations of the outcomes of risky behaviors, which can lead to poor decision-making. These findings match with the concept of a “positive illusion.” Studies investigating functional connectivity within the Default Mode Network (DMN) reveal alterations in individuals with ADHD (Castellanos et al., 2008; Menon, 2011; Fair et al., 2012). The DMN, comprising the medial prefrontal cortex (MPFC), posterior cingulate cortex (PCC), and precuneus, exhibits increased activity during rest or introspection but deactivates during focused tasks (Menon, 2011). In this context, functional connectivity refers to the strength of communication between these brain regions (Matthew et al., 2019). Research suggests weaker connectivity within the MPFC-PCC/precuneus circuit in ADHD compared to non-ADHD individuals (Castellanos et al., 2008; Fair et al., 2012). This pattern is thought to underlie core ADHD symptoms like inattention, impulsivity, and hyperactivity. For instance, the MPFC regulates attention and working memory (Castellanos et al., 2008). Weaker connectivity between the MPFC and PCC/precuneus might hinder the ability to focus and maintain attention on tasks in individuals with ADHD (Castellanos et al., 2008).

Additionally, the PCC is involved in self-awareness and daydreaming, and the precuneus contributes to planning and decision-making (Menon, 2011; Fair et al., 2012). Atypical motivation or poor cognitive control may be the cause of the increased delay (temporal discounting) that is frequently seen in ADHD (Peters and Buchel, 2011). Disrupted communication within this circuit may explain difficulties in monitoring thoughts and behaviors, as well as challenges with planning and choosing appropriate actions, both of which are hallmarks of ADHD. Irregular DMN connectivity is a contributing factor.

## 6 Neurosciences in ADHD

**FIGURE 1:**
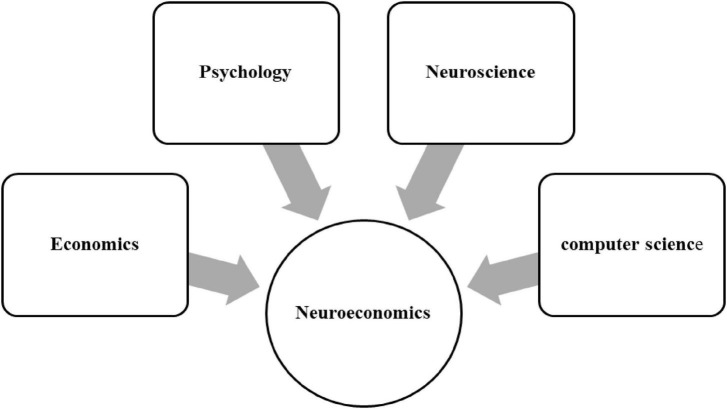
Neuroeconomics combines models, tools, and techniques from economics, psychology, neuroscience, and computer science to gain insights into psychiatric disorders.

Neuroeconomics is a field that studies how social, psychological, and neural factors influence economic decision-making (Rangel et al., 2008). It combines models, tools, and techniques from economics, psychology, neuroscience, and computer science to gain insights into psychiatric disorders (Figure 1). Reinforcement-learning and computational reinforcement learning models have emerged as powerful tools to understand how the brain learns to assign value in different situations and how neuromodulatory systems such as dopamine are disturbed in various mental diseases. These models offer a new language for understanding mental illness and a starting point for connecting detailed neural substrates to behavioral outcomes. They also predict that when a drug, disease, or developmental event perturbs the brain’s capacity to assign appropriate value to behavioral acts or mental states, it can lead to valuation malfunctions.

Researchers are using neuroeconomic approaches to understand better how ADHD affects economic agency, preferences, utilities, and decision-making strategies. Studies have shown that individuals with ADHD may exhibit suboptimal decision-making rather than solely engaging in risky behavior. This means they may choose options with lower values. These choices can vary in complexity and are not just about risk.

The relationship between ADHD and economic behaviors is complex and diverse. Individuals with ADHD may exhibit impulsive spending, risk-taking, time management, and planning challenges, workplace difficulties, and financial aspects of treatment. Understanding these dynamics is crucial for developing tailored interventions and support for therapeutic approaches and broader financial planning and well-being. Managing ADHD often requires therapeutic interventions, medications, and support, which can have an economic impact. By studying an individual’s environment, researchers can gain insights into the decision-making abilities of those with ADHD. People with ADHD tend to engage in suboptimal decision-making. However, it is crucial to differentiate between risky and suboptimal decision-making.

Individuals with ADHD often exhibit structural deviations in their brains, as evidenced by studies using high-resolution fMRI and PET scans. These deviations include hypoactivity in the Prefrontal Cortex (PFC), which plays a crucial role in executive functions, and altered activation patterns in the Striatum, central to reward processing (1). PET studies have also revealed decreased dopamine receptor availability and transporter density in the brains of individuals with ADHD. These anomalies underlie the impulsive and reward-seeking behaviors typical of ADHD. Studies using Event-Related Potentials (ERPs) have shown that individuals with ADHD often exhibit atypical waveforms, suggesting disruptions in attentional processing and subsequent decision-making paradigms. Resting-state fMRI studies have highlighted disrupted functional connectivity patterns in ADHD, particularly between the PFC and other essential decision-making regions. A nuanced understanding of ADHD’s neural architecture can refine therapeutic modalities like Cognitive Behavioral Therapy (CBT), real-time neural data can be utilized to develop neurofeedback interventions, and a detailed understanding of the implicated neural pathways and neurotransmitter systems can guide the development of pharmacological interventions.

Stimulus interference in ADHD has been linked to disturbed activation in ACC, DLPFC, and VLPFC. Similarly, patients with ADHD exhibit hypofunction of medial prefrontal and ventrolateral regions during response interference. During behavioral inhibition, prefrontal dysfunction in patients with ADHD has mainly been associated with hypoactivation in bilateral PFC. Preliminary results suggest that impulsive decision-making in ADHD, as assessed with gambling tasks and risky choice paradigms, may be related to OFC hypofunction. However, neuroimaging studies directly assessing information sampling and proactive inhibition are lacking. Delay discounting has been linked to prefrontal hypofunction in a network comprising ventrolateral and dorsolateral PFC, OFC, and VMPFC). Across different impulse control components, ventrolateral prefrontal dysfunctions may instead be linked to deficient transient, reactive inhibitory processes. In contrast, dorsolateral prefrontal dysfunction may be associated with disturbed sustained task demands, including proactive inhibition and working memory demands in ADHD.

**FIGURE 2:**
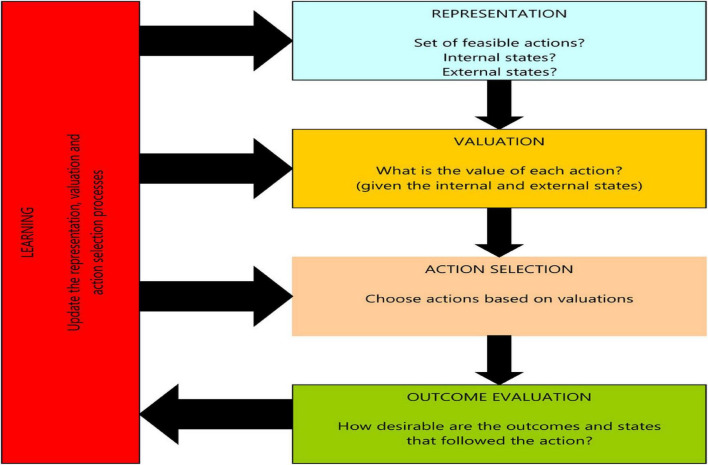
Basic computations involved in the process of making a choice. Value-based decision-making involves five basic processes: constructing a representation of the decision problem, valuing different actions, selecting an action based on its valuation, measuring the desirability of the outcomes, and using outcome evaluation to improve future decisions. Source: Rangel et al. (2008).

The brain has valuation systems. They help people make decisions and respond to situations (Hare et al., 2009). These systems evaluate rewards and risks. They also assess threats and the value of social interactions. They help individuals make decisions based on past experiences and memories. Valuation systems are critical. They shape behavior and guide people toward actions most likely to lead to good outcomes (Platt and Huettel, 2008; Rangel and Hare, 2010). The framework for value-based decision-making has five basic types of computations (Padoa-Schioppa and Conen, 2017). These include how the problem is represented. The value-based decision-making process involves several crucial steps, such as representing the problem, valuing different actions, selecting an action based on its value, rating the outcomes, and using the ratings to improve future decisions. According to the proposed framework, the brain must encode separate value signals at the decision and outcome stages and calculate a value signal for every action under consideration, as shown in Figure 2. Although there are still uncertainties about how these computations match the brain, this system breaks down the decision-making process into manageable parts that can be tested. It helps organize the neuroeconomics literature on the computations being studied and makes predictions about the neurobiology of decision-making. There are uncertainties about how these computations match the brain. However, this system breaks the decision-making process into workable parts. We can test these parts. It organizes the neuroeconomics literature on the computations being studied. It also makes predictions about the neurobiology of decision-making (Camerer et al., 2005).

ADHD has five recognized models. The Behavioral Inhibition/Activation model suggests that children with ADHD have an under-responsive Behavioral Inhibitory System (BIS) and an overactive Behavioral Approach System (BAS). The Energetic model highlights the cognitive, energetic, and executive impact of ADHD. The Executive model links ADHD with deficits in executive function. The Delay aversion model suggests that children with ADHD are often unwilling to delay gratification. The Inhibition model attributes all ADHD-related deficits to a failure of inhibitory control (Rocha et al., 2009).

## 7 Computational model and neuroscience

Computational models use math or algorithms to predict system behavior. In neuroscience, they explain brain functions with four steps: (1) representation, (2) math, (3) simulation, and (4) validation. Models like biophysical and neural networks are essential. They help understand the brain, test ideas, and reduce the need for costly experiments. They also aid in developing treatments. For example, they can analyze the economic behaviors of people with ADHD and suggest tailored support. Complex models and data analysis offer better intervention insights. They use agent-based simulations and machine learning to identify patterns. Additionally, cost-effectiveness analysis helps find the best solutions for individual challenges.

Deterministic computation follows fixed rules for predictable outcomes, like basic math. It gives exact results based on set parameters. On the other hand, probabilistic models offer outcomes as probabilities from a range of results. They are faster and simpler than non-deterministic models (David et al., 2005). These models are suitable for predicting outcomes with known parameters. Non-deterministic computation, like in machine learning, is more complex. It can produce unpredictable results, influenced by various factors. Poor training data can make these models less accurate. Advanced techniques, such as model training and adaptation, help manage errors.

Deterministic computation in neural terms is about simple, predictable functions. It involves fixed pathways, similar to essential brain functions. For example, reflex arcs are simple pathways that trigger immediate, predictable responses. Non-deterministic computation in the brain is more complex. It includes adaptive, interconnected networks. These networks change based on various factors, just like machine learning models. They lead to outcomes influenced by context, past experiences, and ongoing processes. Processes like decision-making and learning involve non-deterministic pathways. The brain’s ability to adapt and learn, or neural plasticity, depends on sensory input and experiences. Strategies like cognitive training aim to reduce errors in brain processing. They help improve neural pathways and overall brain function. The brain’s pathways can be seen as both deterministic and non-deterministic. This view is crucial for understanding human cognition.

## 8 Neuroeconomic modeling and ADHD

The relationship between ADHD and decision-making involves various indices, brain areas, and measures. These include impulsivity, risk-taking, time perception and planning, and response inhibition. Impulsivity is measured through questionnaires or tasks requiring immediate and delayed rewards. Risk-taking is assessed through gambling tasks or other risk-reward evaluations. Time estimation tasks or long-term planning assessments measure time perception and planning. Response inhibition is evaluated using Stroop or Go/No-Go tasks.

The neuroscientific evidence indicated that the well-known adolescent behavioral immaturity was not simply due to poor choices or different values but was at least partly due to factors that were not entirely under their control; that is, brain immaturity was based upon findings supporting what is alternately termed the dual systems (DS) model or the imbalance model of the adolescent brain. According to these models, risky, impulsive, sensation-seeking behavior in adolescence is the product of an interaction between two distinct brain circuits: the cognitive control and reward systems. The DS model posits a differential development in the adolescent brain of these two brain systems, which influence self-control. The reward brain circuit undergoes rapid and dramatic development around puberty. It leads to striking increases in impulsivity, sensation seeking, and risk-taking, which remains high into the early twenties when it begins to decline. This heightened tendency to act impulsively is significantly amplified in emotionally charged situations where decisions are required in the ‘heat of the moment.’ However, the cognitive control brain circuit is still developing and does not mature until the early twenties. Due to the imbalance between these two brain circuits, adolescents have been described as being ‘all gasoline, no brakes.’ Although this characterization is an obvious exaggeration and oversimplification, neuroscience provides compelling evidence for a biological basis for ‘what every parent knows’; that is, adolescence is a time of diminished capacity for self-control as compared to adults, in part, because of an imbalance between these two systems (Robert, 2016).

Recent research has identified three brain networks that could be linked to ADHD. The first network connects the medial prefrontal cortex and posterior cingulate cortex - the default mode network. Altered connectivity patterns within this network can disrupt the ability to order utilities, prospect about desired future states, set future goals, and implement aims. The second network is associated with the dorsal frontostriatal, which includes the dorsolateral prefrontal cortex and dorsal striatum. Deficits in this network can cause executive dysfunction, leading to difficulty comparing outcome options and making choices. Finally, the third network is responsible for dopaminergic dysregulation in a ventral frontostriatal network, encompassing the orbitofrontal cortex, ventral striatum, and amygdala. This network can disrupt the processing of cues for future utility, evaluating experienced outcomes (feedback) and learning associations between cues and outcomes (Sonuga-Barke and Fairchild, 2012).

ADHD is frequently linked to specific regions of the brain that can affect decision-making processes. These areas include the prefrontal cortex (PFC), responsible for executive functions such as planning, impulse control, and decision-making. On the other hand, the striatum plays a critical role in reward processing and may affect impulsivity and risk-taking. Additionally, the anterior cingulate cortex (ACC) is associated with error monitoring and adaptive behavior, which can influence response inhibition, and the dorsolateral prefrontal cortex (DLPFC) is involved in working memory and cognitive control, which can impact complex decision-making tasks (Arnsten, 2009).

While older ideas about executive control have helped describe the phenomenology of control, future progress will require more computational approaches as only through such models can competing ideas be differentiated. Modeling efforts have already been applied to executive control and decision-making in humans. Simple choice, a fundamental aspect of economic decision-making, involves weighing the pros and cons of various options and deciding based on that evaluation. Understanding how simple choices work can provide valuable insights into human behavior and decision-making processes.

## 9 Psychiatric disorders

The decision-making processes of the human brain can exhibit behaviors and perceptions that diverge significantly from societal norms, leading to the classification of such deviations as psychiatric disorders. The medical community categorizes mental illnesses based on well-established diagnostic criteria primarily focused on observable behavioral features. Despite this emphasis, there exists a substantial body of biological data that directly influences psychiatric conditions, including insights from animal models of nicotine addiction, anxiety, depression, and schizophrenia that have contributed extensive knowledge on neurotransmitter systems, receptors, and gene expression patterns. However, there remains a limited understanding of how psychotropic drugs impact the brain’s decision-making and perceptual mechanisms despite their known effects on neuromodulatory systems and resultant behavioral changes. This presents an opportunity for fields like neuroeconomics and computational sciences to bridge the gap between biological knowledge and behavioral manifestations in psychiatric disorders.

Disturbances within the reward system have been documented across major psychiatric disorders, including substance use disorders (SUD), which exhibit effects akin to those produced by natural rewards. For instance, addicted individuals often display hyper-responsivity to relevant cues within the mesolimbic reward system, while chronic stimulant users show down-regulation of striatal D2 receptor density. Understanding the pathophysiology of reward is critical, as it has led to reevaluating pathological gambling (PG) as a nonsubstance-related addictive disorder due to similarities with addictive behaviors. PG shares similarities in reward pathophysiology with mood disorders, showing blunted activation of the mesolimbic-prefrontal cortex in response to nonspecific rewards but increased activation when exposed to gambling-related stimuli. Major depressive disorder (MDD) is characterized by reduced motivation to obtain rewards and diminished pleasure from rewarding experiences, potentially linked to abnormal dopamine-mediated responses involving mesolimbic dopaminergic projections.

Schizophrenia also exhibits abnormal dopamine responses to rewarding stimuli, particularly in orbital and dorsal prefrontal structures. ADHD is recognized as an etiological factor influencing reward-related processes. Neuroeconomics offers promise in addressing gaps related to reward processing in psychiatric disorders and elucidating how neural systems interact to develop or sustain these conditions.

Research on ADHD and neuroeconomics has explored how cognitive challenges associated with ADHD impact economic decision-making. Individuals with ADHD may demonstrate differences in reward processing, risk assessment, and impulsive decision-making, affecting economic choices such as susceptibility to immediate rewards and tendencies toward impulsive financial decisions. However, research findings vary due to significant individual differences among people with ADHD, influenced by co-occurring conditions and personal experiences. Further research integrating neuroscience, psychology, and economics is essential for a detailed understanding of the neuroeconomic aspects of ADHD and developing interventions to support informed and adaptive economic decision-making in affected individuals.

## 10 Implications

### 10.1 Future implications

ADHD can cause impulsive spending habits, leading to financial instability. It can also affect investment decisions by seeking high risk or fearing failure or loss. ADHD symptoms may impact career choices, job performance, and long-term earning potential, leading to additional healthcare costs that can significantly affect individual or family finances. Specialized educational support for students with ADHD can entail extra costs for families and educational systems. ADHD may affect academic achievement and influence future earning potential and economic well-being. Challenges in focus and task management may affect workplace productivity. Implementing workplace accommodations for employees with ADHD may involve costs. However, it can lead to long-term productivity and employee well-being benefits. Treatment and support for individuals with ADHD represent a significant cost in the healthcare system. Some individuals with ADHD might require additional social support, impacting social welfare systems. Understanding the economic behaviors of individuals with ADHD can lead to targeted interventions and support. Cost-benefit analyses might help policymakers evaluate the economic efficiency of interventions and support systems for ADHD.

Understanding the relationship between ADHD and economic behaviors can lead to targeted interventions such as financial planning, budgeting strategies, or therapeutic approaches that consider economic decision-making. Financial planners working with ADHD clients might incorporate strategies to manage impulsivity in spending or support long-term financial planning. The intersection of ADHD with economic behaviors represents a vital area of study and practice, reflecting the complex, layered nature of ADHD in real-world contexts. It is essential to comprehend these dynamics to tailor interventions and support regarding therapeutic approaches and broader financial planning and well-being.

### 10.2 Policy implications

(1) ADHD imposes a significant financial burden on individuals and society, emphasizing the need for policymakers to prioritize early diagnosis, treatment, and support services. This requires ensuring access to affordable healthcare, educational resources, and behavioral interventions for those in need.
(2) To address the unique educational challenges faced by children with ADHD, schools must adopt inclusive policies catering to diverse learning needs providing appropriate resources for affected students.
(3) Encouraging workplaces to adopt policies that support individuals with ADHD can lead to increased productivity and job satisfaction. Accommodations such as flexible work schedules, task management strategies, and employer training programs can be implemented.
(4) Public awareness campaigns can reduce the stigma associated with ADHD, promoting acceptance and understanding within communities. This can increase support and access to services for those affected.
(5) Interdisciplinary research initiatives that bridge neuroscience, economics, and public health are essential to developing targeted interventions and policies based on a nuanced understanding of ADHD.

The insights gained from neuroeconomics have practical implications across various domains, including marketing, public policy, and finance. For instance, marketers can better understand how consumers respond to pricing strategies or advertising techniques based on their neural responses. Policymakers can design more effective interventions to promote pro-social behaviors and reduce economic disparities. Financial institutions can develop strategies that align with the cognitive biases of investors. However, it is essential to recognize that “opening the black box” of the human brain is an ongoing and complex endeavor (Raj, 2023).

## 11 Conclusion

ADHD is a significant public health challenge that affects both children and adults globally. It has various economic implications, including academic underachievement, substance abuse, antisocial behavior, and increased rates of accidents and hospitalizations, leading to societal costs. The economic burden of ADHD is complex, including direct costs like medication and therapy, indirect costs such as missed work due to caregiver strain, and intangible costs like marital stress and disruptions in education and extracurricular activities. Neuroeconomic studies explore how ADHD influences economic decision-making, focusing on impulsivity, risk-taking, and reward processing. Individuals with ADHD may exhibit suboptimal decision-making, choosing options that do not maximize expected value. Specific brain regions affected by ADHD, such as the prefrontal cortex (PFC), striatum, and anterior cingulate cortex (ACC), play crucial roles in executive functions, reward processing, and response inhibition, impacting decision-making abilities. The intersection of ADHD with economic behaviors underscores the importance of broad research integrating neuroscience, psychology, and economics to develop effective interventions and support strategies.

## Author contributions

AC: Conceptualization, Project administration, Resources, Supervision, Writing – original draft, Writing – review and editing. MS: Writing – review and editing, Project administration, Conceptualization.
